# Transcriptional Induction of Cystathionine γ-Lyase, a Reactive Sulfur-Producing Enzyme, by Copper Diethyldithiocarbamate in Cultured Vascular Endothelial Cells

**DOI:** 10.3390/ijms21176053

**Published:** 2020-08-22

**Authors:** Tomoya Fujie, Akane Takahashi, Musubu Takahashi, Takato Hara, Asuka Soyama, Kosho Makino, Hideyo Takahashi, Chika Yamamoto, Yoshito Kumagai, Hiroshi Naka, Toshiyuki Kaji

**Affiliations:** 1 Faculty of Pharmaceutical Sciences, Toho University, 2-2-1 Miyama, Funabashi 274-8510, Japan; t-fujie@phar.toho-u.ac.jp (T.F.); takato.hara@phar.toho-u.ac.jp (T.H.); yamamoto@phar.toho-u.ac.jp (C.Y.); 2 Faculty of Pharmaceutical Sciences, Tokyo University of Science, 2641 Yamazaki, Noda 278-8510, Japan; 3B18556@alumni.tus.ac.jp (A.T.); 3a19703@ed.tus.ac.jp (M.T.); j3a10050@ed.tus.ac.jp (A.S.); kosho-maki@rs.tus.ac.jp (K.M.); hide-tak@rs.tus.ac.jp (H.T.); 3 Environmental Biology Laboratory, Faculty of Medicine, University of Tsukuba, 1-1-1 Tennodai, Tsukuba, Ibaraki 305-8575, Japan; yk-em-tu@md.tsukuba.ac.jp; 4 Research Center for Materials Science, Nagoya University, Furo-cho, Chikusa-ku, Nagoya 464-8602, Japan

**Keywords:** endothelial cell, copper diethyldithiocarbamate, cystathionine γ-lyase, mitogen-activated protein kinase, hypoxia-inducible factor-1, bio-organometallics

## Abstract

As toxic substances can enter the circulating blood and cross endothelial monolayers to reach parenchymal cells in organs, vascular endothelial cells are an important target compartment for such substances. Reactive sulfur species protect cells against oxidative stress and toxic substances, including heavy metals. Reactive sulfur species are produced by enzymes, such as cystathionine γ-lyase (CSE), cystathionine β-synthase, 3-mercaptopyruvate sulfurtransferase, and cysteinyl-tRNA synthetase. However, little is known about the regulatory mechanisms underlying the expression of these enzymes in vascular endothelial cells. Bio-organometallics is a research field that analyzes biological systems using organic-inorganic hybrid molecules (organometallic compounds and metal coordinating compounds) as molecular probes. In the present study, we analyzed intracellular signaling pathways that mediate the expression of reactive sulfur species-producing enzymes in cultured bovine aortic endothelial cells, using copper diethyldithiocarbamate (Cu10). Cu10 selectively upregulated CSE gene expression in vascular endothelial cells independent of cell density. This transcriptional induction of endothelial CSE required both the diethyldithiocarbamate scaffold and the coordinated copper ion. Additionally, the present study revealed that ERK1/2, p38 MAPK, and hypoxia-inducible factor (HIF)-1α/HIF-1β pathways mediate transcriptional induction of endothelial CSE by Cu10. The transcription factors NF-κB, Sp1, and ATF4 were suggested to act in constitutive CSE expression, although the possibility that they are involved in the CSE induction by Cu10 cannot be excluded. The present study used a copper complex as a molecular probe to reveal that the transcription of CSE is regulated by multiple pathways in vascular endothelial cells, including ERK1/2, p38 MAPK, and HIF-1α/HIF-1β. Bio-organometallics appears to be an effective strategy for analyzing the functions of intracellular signaling pathways in vascular endothelial cells.

## 1. Introduction

Blood vessels ubiquitously exist in human organs. Vascular endothelial cells cover the luminal surfaces of blood vessels in monolayers that serve as barriers between the blood and the parenchymal cells of the organs. Toxic substances that enter the circulating blood have to cross these endothelial monolayers to reach the parenchymal cells. In other words, vascular endothelial cells are an important defense against such toxic substances. We studied the vascular toxicology of heavy metals using a vascular endothelial cell culture system and found that cadmium and lead cause specific functional damage to these cells. For example, cadmium causes morphological damage to the endothelial cell monolayer [1] and reduces endothelial cell mediated fibrinolytic activity by inducing plasminogen activator inhibitor type 1 [2]. Lead also reduces fibrinolytic activity; however, the mechanism is suppression of tissue type plasminogen activator expression [3]. Interestingly, vascular endothelial cells are protected from cadmium cytotoxicity by zinc [4] without induction of metallothionein, a cytoprotective protein against heavy metal toxicity [5]; zinc mediated protection is due to a decrease in intracellular cadmium accumulation [6]. Metallothionein cannot be induced by zinc, a typical inducer, in vascular endothelial cells [7]. In the cytosol of zinc treated cells, the metal accumulates in the low-molecular-mass fraction [6], indicating that low-molecular mass molecules sequester a cytosolic zinc pool.

We focused our attention on reactive sulfur species, such as hydrogen sulfide, cysteine persulfide, and glutathione persulfide, because they are of low molecular mass and are highly nucleophilic [8,9]. These characteristics of reactive sulfur species are consistent with those of the putative zinc pool in vascular endothelial cells. The enzymes, such as cystathionine γ-lyase (CSE) [10], cystathionine β-synthase (CBS) [10], 3-mercaptopyruvate sulfurtransferase (3-MST) [11], and cysteinyl-tRNA synthetase (CARS2) [12] produce reactive sulfur species. Levels of reactive sulfur species appear to depend on the expression of these enzymes; however, little is known about the regulatory mechanisms underlying their expression. Clarification of the intracellular signaling pathways that mediate the expression of reactive sulfur species-producing enzymes will contribute to our understanding of zinc metabolism and heavy metal toxicity in vascular endothelial cells. Additionally, such studies will contribute to our understanding of cellular defense mechanisms, because reactive sulfur species are protective against oxidative stress [10].

Bio-organometallics is a research field that analyzes biological systems using organic-inorganic hybrid molecules (organometallic compounds and coordinating compounds) as molecular probes [13]. We characterized the cytotoxicity of the hybrid molecules and applied this strategy to studies of intracellular signaling pathways that mediate regulation of vascular endothelial cell functions. Organic-inorganic hybrid molecules exhibit unique cytotoxicity in vascular endothelial cells [14,15,16,17,18]. Specifically, the cytotoxicity of organic-inorganic hybrid molecules is not simply dependent on their hydrophobicity, intracellular accumulation, molecular structures, or the intramolecular metals they contain. Copper diethyldithiocarbamate (Cu10) was found to be an activator of Nrf2 from a library of organic-inorganic hybrid molecules in cultured vascular endothelial cells [19]. Cu10 has been found to induce endothelial metallothionein, a cytoprotective protein. Subsequent studies using this copper complex have revealed that metallothionein induction is mediated by different intracellular signaling pathways for different metallothionein isoforms [20,21,22]. Proteoglycans synthesized by vascular endothelial cells play important roles in regulating the growth factor/cytokine activity [23] and blood-coagulation [24]. Bio-organometallics studies have revealed that hypoxia-inducible factor-1α (HIF-1α)/HIF-1β [25] and p38 mitogen-activated phosphate kinase (MAPK) [26] pathways can mediate expression of syndecan-4, a transmembrane heparan sulfate proteoglycan found in vascular endothelial cells. More recently, an elegant prodrug approach for generating copper diethyldithiocarbamate in prostate cancer cells has been developed using prochelators activated by γ-glutamyl transferase or by prostate specific antigens [27,28].

Several reports examine the conditions in which reactive sulfur-producing enzymes, especially CSE, are induced in vascular endothelial cells. For example, hypoxia increases the protein levels of HIF-1α, which activates the Nox-4-heme-regulated inhibitor (HRI) kinase)-eIF2α- activating transcription factor 4 (ATF4) pathway to induce CSE expression in human umbilical vein endothelial cells [29]. In rat aortic endothelial cells, calcium-sensing receptors elevate the expression of CSE in a phospho-calmodulin kinases II-dependent manner to inhibit platelet activation [30]. One week of protein-restricted diet in mice increased CSE expression, with a corresponding decrease in vein graft disease, suggesting ATF4 involvement in this expression [31]. Beyond these studies, little is known about the intracellular signaling pathways that mediate the expression of reactive sulfur species-producing enzymes. The present study was undertaken to reveal bio-organometallic sensitive pathways mediating endothelial cell CSE expression.

## 2. Results

### 2.1. Transcriptional Induction of CSE, a Reactive Sulfur Species-Producing Enzyme

**Figure 1:**
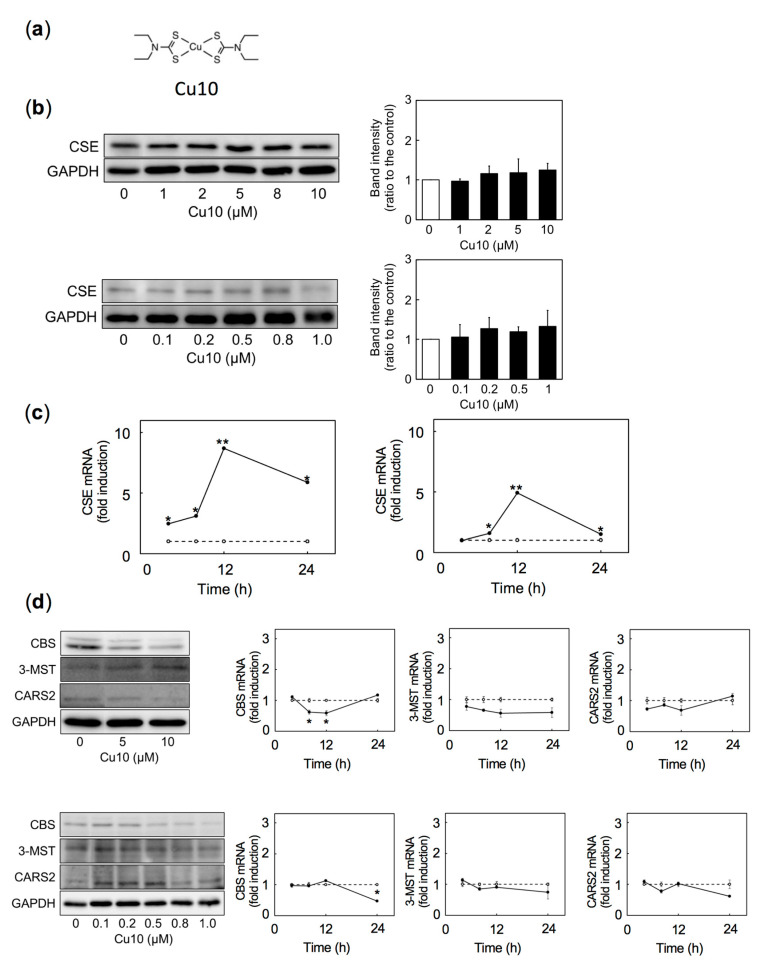
Expression of reactive sulfur species-producing enzymes in dense and sparse vascular endothelial cells after treatment with copper diethyldithiocarbamate (Cu10). (**a**) Structure of Cu10. (**b**) Levels of cystathionine γ-lyase (CSE) protein in dense (upper panels) and sparse (lower panels) cell cultures after treatment with Cu10. The ratio to the intensity of CSE to that of glyceraldehyde-3-phosphate dehydrogenase (GAPDH); values are mean ± SE of three replicates from three independent experiments. (**c**) Levels of CSE mRNA in dense (left panel) and sparse (right panel) cell cultures after treatment with Cu10. (**d**) Levels of cystathionine β-synthase (CBS), 3-mercaptopyruvate sulfurtransferase (3-MST), and cysteinyl-tRNA synthetase (CARS2) protein (left panels) and mRNA (right panels) in dense (upper panels) and sparse (lower panels) cell cultures after treatment with Cu10. The results of the densitometric analysis are shown in Figure S1. Dense cultures of bovine aortic endothelial cells were treated with Cu10 (1, 2, 5, 8, or 10 µM) for 24 h (left panels) or with Cu10 (5 µM) for 4, 8, 12, and 24 h (right panels). ○, Control, ●, Cu10 treatment. Values are mean ± SE of three technical replicates. Significant difference from corresponding control, * *p* < 0.05; ** *p* < 0.01. Sparse cultures of bovine aortic endothelial cells were treated with Cu10 (0.1, 0.2, 0.5, 0.8, or 1.0 µM) for 24 h (left panels) or with Cu10 (5 µM) for 4, 8, 12, and 24 h (right panels). ○, Control, ●, Cu10 treatment. Values are mean ± SE of three technical replicates. Significant difference from corresponding control, * *p* < 0.05; ** *p* < 0.01.

Figure 1 shows expression of CSE, CBS, 3-MST, and CARS2 in vascular endothelial cells treated with or without Cu10. The structure of Cu10 is shown in Figure 1a. Because the response of vascular endothelial cells to exogenous factors is often cell density-dependent [32,33,34,35,36], both dense and sparse cultures of the cells were used in this experiment. CSE protein expression was not altered by Cu10 in dense (Figure 1b, upper panels) or sparse cultures (Figure 1b, lower panels). By contrast, levels of CSE mRNA were elevated by Cu10 after 4 h or longer in a time-dependent manner in both dense (Figure 1c, left panel) and sparse cultures (Figure 1c, right panel). Expression of the other reactive sulfur species-producing enzymes—CBS, 3-MST, and CARS2—were not elevated by Cu10 at protein (Figure 1d, left panels) or mRNA (Figure 1d, right panels) levels in dense (Figure 1d, upper panels) or sparse (Figure 1d, lower panels) cultures. These results suggest that Cu10 selectively stimulates transcription of CSE without changing CSE protein expression, regardless of cell density in vascular endothelial cells. These results also suggest that Cu10 may serve as a molecular probe to analyze intracellular signaling pathways that mediate endothelial CSE gene expression.

### 2.2. Role of Copper and Ligand Structure of the Cu10 Molecule in Induction of Endothelial CSE Transcription

**Figure 2:**
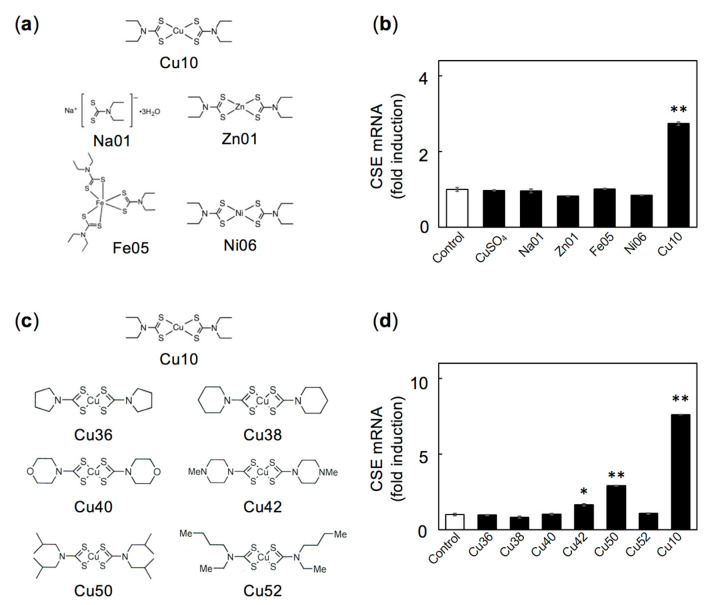
CSE mRNA level in vascular endothelial cells after treatment with Na01, Zn01, Ni06, CuSO_4_, or Cu10 analogs. (**a**) Structures of Cu10, Na01, Zn01, Fe05, and Ni06. (**b**) CSE mRNA level. Dense cultures of bovine aortic endothelial cells were incubated with CuSO_4_, Na01, Zn01, Fe05, Ni06, or Cu10 (5 µM each) for 12 h. Values are mean ± SE of three technical replicates. ** Significant difference from the control, *p* < 0.01. (**c**) Structure of Cu36, Cu38, Cu40, Cu42, Cu50, and Cu52. (**d**) CSE mRNA level. Dense cultures of bovine aortic endothelial cells were treated with Cu36, Cu38, Cu40, Cu42, Cu50, Cu52, or Cu10 (5 µM each) for 12 h. Values are mean ± SE of three technical replicates. Significant difference from control, * *p* < 0.05; ** *p* < 0.01.

To examine whether the copper atom in Cu10 is critical to inducing the transcription of endothelial cell CSE, we investigated the effects of zinc, iron, and nickel complexes with the same diethyldithiocarbamate ligand (Figure 2a) on CSE mRNA. Although Cu10 significantly increased CSE mRNA, neither copper sulfate nor Na01 (sodium diethyldithiocarbamate trihydrate, the ligand portion of Cu10) affected CSE mRNA levels (Figure 2b), suggesting that the complete Cu10 complex, including the copper atom and the diethyldithiocarbamate is required to induce transcription of CSE in vascular endothelial cells. In addition, neither zinc(II) bis(diethyldithiocarbamate) (Zn01), iron(III) tris(diethyldithiocarbamate) (Fe05), or nickel(II) bis(diethyldithiocarbamate) (Ni06) increased CSE mRNA levels (Figure 2b), indicating that the copper in Cu10 is critical for inducing transcription of CSE in endothelial cells.

The effects of Cu10 analogs (Figure 2c) on CSE mRNA levels in vascular endothelial cells were investigated to evaluate the importance of the diethyldithiocarbamate structure for inducing transcription of endothelial CSE. As shown in Figure 2d, Cu10 markedly increased levels of endothelial cell CSE mRNA; however, the Cu10 analogs—bis(1-pyrrolidinecarbodithioato)copper (II) (Cu36), bis(1-piperidinecarbodithioato)copper (II) (Cu38), bis(4-morpholinecarbodithioato)copper (II) (Cu40), and bis(butylethyldithiocarbamato)copper (II) (Cu52)—failed to exhibit similar activity, although bis(4-methylpiperazine-1-carbodithioato)copper (II) (Cu42) and bis(diisobutyldithiocarbamato)copper (II) (Cu50) weakly increased CSE mRNA. This suggests that the copper and the diethyldithiocarbamate structure are important for inducing endothelial cell CSE gene expression.

### 2.3. The extracellular signal-regulated kinase (ERK) and p38 MAPK Pathways are Involved in Cu10-Mediated Induction of CSE Expression in Endothelial Cells

**Figure 3:**
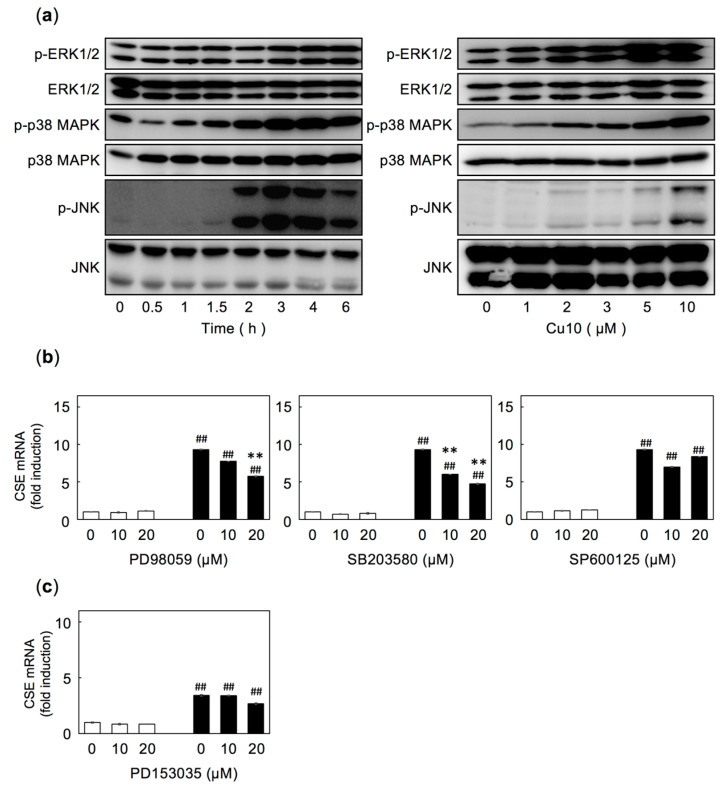
Possible involvement of the mitogen-activated phosphate kinase (MAPK) pathway in inducing CSE transcription with Cu10. (**a**) Phosphorylation of extracellular signal-regulated kinase 1/2 (ERK1/2), p38 MAPK, and c-jun N-terminal kinase (JNK) in vascular endothelial cells after treatment with Cu10. Dense cultures of bovine aortic endothelial cells were treated with Cu10 (5 µM) for 0.5, 1, 1.5, 2, 3, 4, and 6 h (left panel) or with Cu10 (1, 2, 3, 5, and 10 µM) for 3 h (right panel). The results of the densitometric analysis were shown in Figure S2. (**b**) Possible involvement of MAPK pathways in the induction of CSE transcription in vascular endothelial cells treated with Cu10. Dense cultures of bovine aortic endothelial cells were pretreated with the ERK1/2 inhibitor PD98059, the p38 MAPK inhibitor SB203580, or the JNK inhibitor SP600125 (10 and 20 µM each) for 3 h, then treated with (■) or without (□) Cu10 (5 µM) for 12 h. ** Significant difference from the corresponding control, *p* < 0.01. ^##^ Significant difference from the corresponding vehicle control (no Cu10), *p* < 0.01. (**c**) Possible involvement of epidermal growth factor receptor (EGFR) in the induction of CSE transcription in vascular endothelial cells treated with Cu10. Dense cultures of bovine aortic endothelial cells were pretreated with the EGFR inhibitor PD153035 (10 and 20 µM) for 3 h, then treated with (■) or without (□) Cu10 (5 µM) for 12 h. Values are mean ± SE of three technical replicates. ^##^ Significant difference from the corresponding vehicle control (no Cu10), *p* < 0.01.

As we recently reported that Cu10 activates the p38 MAPK pathway in vascular endothelial cells [26], we investigated the role of the MAPK pathways in Cu10-mediated induction of endothelial CSE transcription (Figure 3). We confirmed that Cu10 activates MAPKs (extracellular signal-regulated kinase (ERK), p38 MAPK, and c-jun N-terminal kinase (JNK)) in vascular endothelial cells in a time- and dose-dependent manner (Figure 3a), suggesting that MAPK pathways may be involved in the induction of CSE transcription by Cu10. To examine this possibility, vascular endothelial cells were pretreated with the ERK inhibitor PD98059, the p38 MAPK inhibitor SB203580, or the JNK inhibitor SP600125, then treated with Cu10 (Figure 3b). Induction of CSE transcription by Cu10 was significantly suppressed by either PD98059 or SB203580 (Figure 3b, left and middle panels), indicating that the ERK and p38 MAPK kinase pathways are involved in the induction of endothelial CSE transcription by Cu10. However, Cu10 induced CSE transcription was unaffected by SP600125 (Figure 3b, right panel), suggesting that the JNK pathway is not involved in induction by Cu10.

As we recently reported that the epidermal growth factor receptor (EGFR) is one of the upstream molecules involved in activating p38 MAPK by electrophilic metal compounds, such as methylmercury in vascular endothelial cells [37], we assessed the involvement of EGFR in Cu10-increased CSE mRNA in endothelial cells. Pretreatment with the EGFR inhibitor PD153035 failed to suppress Cu10-mediated induction of CSE mRNA in these cells (Figure 3c), suggesting that the activation of the ERK and p38 MAPK pathways that mediate Cu10-induced CSE transcription is not downstream of EGFR in vascular endothelial cells.

### 2.4. HIF-1α/HIF-1β is Also Involved in Cu10-Mediated Induction of Endothelial Cell CSE Transcription

**Figure 4:**
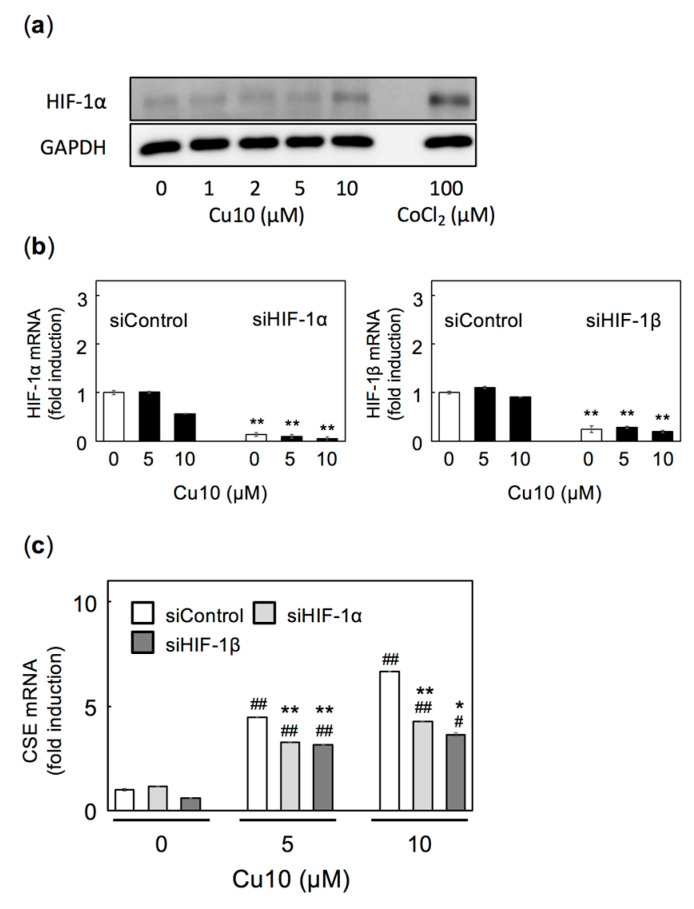
Possible involvement of hypoxia signaling in induction of CSE transcription by Cu10. (**a**) hypoxia-inducible factor (HIF)-1α activation by Cu10. Dense cultures of bovine aortic endothelial cells were treated with Cu10 (1, 2, 5, and 10 µM) or CoCl_2_ (100 µM) for 3 h. CoCl_2_ was used as a positive control. The results of the densitometric analysis are shown in Figure S3. (**b**) HIF-1α and HIF-1β mRNAs in vascular endothelial cells after siRNA-mediated knockdown of HIF-1α and HIF-1β, respectively. Subconfluent cultures of bovine aortic endothelial cells transfected with either the control, HIF-1α, or HIF-1β siRNA for 12 h and treated with Cu10 (5 and 10 µM) for 12 h. Values are mean ± SE of three technical replicates. ** Significant difference from the corresponding siControl, *p* < 0.01. (**c**) Possible involvement of HIF-1α and HIF-1β in the induction of CSE transcription in vascular endothelial cells after Cu10 treatment. Subconfluent cultures of bovine aortic endothelial cells transfected with either the control, HIF-1α, or HIF-1β siRNA for 12 h and treated with Cu10 (5 and 10 µM) for 12 h. Values are mean ± SE of three technical replicates. Significant difference from the corresponding siControl, * *p* < 0.05; ** *p* < 0.01. Significant difference from the corresponding vehicle treated control (no Cu10), ^#^ *p* < 0.05; ^##^ *p* < 0.01.

Under hypoxic conditions, prolyl hydroxylase activity-dependent degradation of HIF-1α is inhibited, increasing HIF-1α protein levels; HIF-1α is transported into the nuclei, where it forms a complex with HIF-1β and functions as a transcription factor. It is possible that HIF-1α is stabilized by Cu10-mediated inhibition of the ubiquitin-proteasome system [19] and is thus involved in Cu10′s induction of CSE transcription in vascular endothelial cells. As some reports suggest that hypoxia increases CSE expression in mammalian cells other than vascular endothelial cells [29,38], it is possible that HIF-1α and HIF-1β can induce endothelial cell CSE transcription. Cu10 slightly increases HIF-1α protein expression (Figure 4a) without changing levels of HIF-1α mRNA (Figure 4b, left panel). HIF-1α is upregulated independent of mRNA level, but siRNA can suppress the upregulation of HIF-1α [39]. siRNA-mediated knockdown of either HIF-1α or HIF-1β (Figure 4b, left and middle panels) partly, but significantly, suppressed Cu10-induced endothelial cell CSE transcription (Figure 4c). These results suggest that hypoxia signaling, mediated by HIF-1α/HIF-1β, is also involved in the induction of endothelial cell CSE transcription by Cu10.

**Figure 5:**
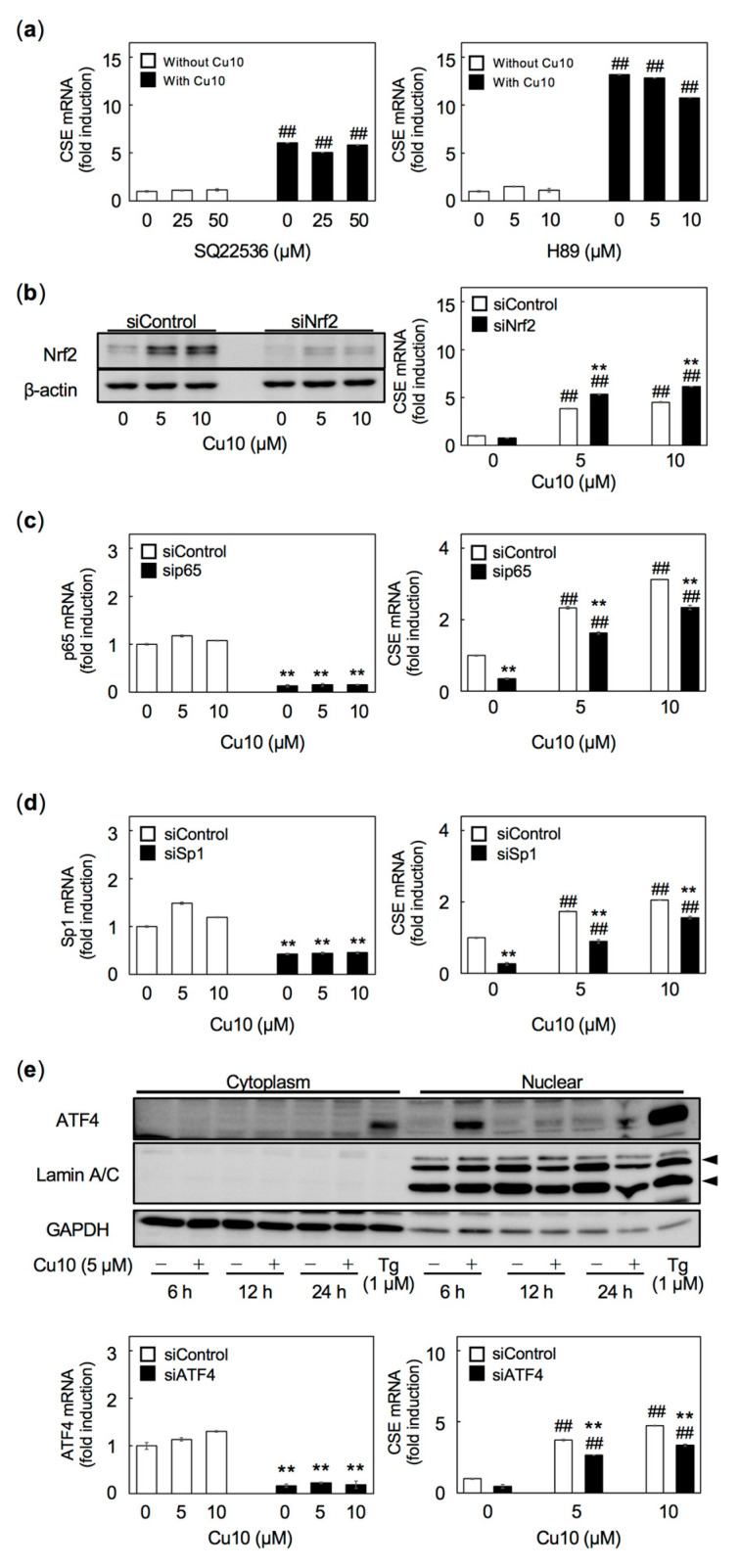
Possible involvement of cyclic AMP mediated signaling and transcription factors—Nrf2, NFκB, Sp1, and ATF4—in the induction of CSE transcription by Cu10. (**a**) Possible involvement of cyclic AMP in the induction of CSE transcription in vascular endothelial cells treated with Cu10. Densely cultured bovine aortic endothelial were pretreated with the adenylate cyclase inhibitor SQ22536 (25 and 50 µM) (left panel) or the protein kinase A inhibitor H89 (5 and 10 µM) (right panel) for 1 h, then treated with (■) or without (□) Cu10 (5 µM) for 12 h. Values are mean ± SE of three samples. ^##^Significant difference from the corresponding vehicle control (no Cu10), *p* < 0.01. (**b**) Possible involvement of Nrf2 in the induction of CSE transcription in vascular endothelial cells treated with Cu10. Nrf2 protein expression (left panel). Confluent cultures of bovine aortic endothelial cells were treated with Cu10 (5 and 10 µM) for 6 h. The results of the densitometric analysis are shown in Figure S4. CSE mRNA level (right panel). Subconfluent cultures of bovine aortic endothelial cells were transfected with control or Nrf2 siRNA for 4 h and treated with (■) or without (□) Cu10 (5 and 10 µM) for 12 h. Values are mean ± SE of three technical replicates. ** Significant difference from the corresponding siControl, *p* < 0.01. ^##^ Significant difference from the corresponding vehicle control (no Cu10), *p* < 0.01. (**c**) Possible involvement of NF-κB in the induction of CSE transcription in vascular endothelial cells treated with Cu10. p65 mRNA (left panel) and CSE mRNA (right panel). Subconfluent cultures of bovine aortic endothelial cells were transfected with the control or p65 siRNA for 6 h and treated with or without Cu10 (5 and 10 µM) for 12 h. Values are mean ± SE of three technical replicates. ** Significant difference from the corresponding siControl, *p* < 0.01. ^##^ Significant difference from the corresponding vehicle control (no Cu10), *p* < 0.01. (**d**) Possible involvement of Sp1 in the induction of CSE transcription in vascular endothelial cells treated with Cu10. p65 mRNA (left panel) and CSE mRNA (right panel). Subconfluent cultures of bovine aortic endothelial cells were transfected with control or Sp1 siRNA for 6 h and treated with or without Cu10 (5 and 10 µM) for 12 h. Values are mean ± SE of three technical replicates. ** Significant difference from the corresponding siControl, *p* < 0.01. ^##^ Significant difference from the corresponding vehicle control (no Cu10), *p* < 0.01. (**e**) Possible involvement of ATF4 in the induction of CSE transcription in vascular endothelial cells treated with Cu10. Expression of the ATF4 protein (upper panel). Dense cultures of bovine aortic endothelial cells were treated with or without Cu10 (5 µM) for 6, 12, and 24 h (upper panel). Thapsigargin (Tg) was used as a positive control. The results of the densitometric analysis are shown in Figure S5. ATF4 mRNA (lower left panel) and CSE mRNA (lower right panel). Subconfluent cultures of bovine aortic endothelial cells were transfected with the control or ATF4 siRNA for 4 h and treated with or without Cu10 (5 and 10 µM) for 12 h (lower panels). Values are mean ± SE of three technical replicates. ** Significant difference from the corresponding siControl, *p* < 0.01. ^##^ Significant difference from the corresponding vehicle control (no Cu10), *p* < 0.01.

We also examined the protein kinase A pathway as a possible mediator of CSE transcription [40]. Neither the adenylate cyclase inhibitor SQ22536 nor the protein kinase A inhibitor H89 affected the induction of endothelial cell CSE transcription by Cu10 (Figure 5a). Similarly, siRNA-mediated knockdown of the transcription factor Nrf2 failed to suppress the induction of CSE transcription by Cu10 (Figure 5b). siRNA-mediated knockdown of the NF-κB components p65 (Figure 5c), Sp1 (Figure 5d), and ATF4 (Figure 5e) reduced CSE mRNA levels in the presence or absence of Cu10, suggesting that these transcriptional factors are involved in constitutive gene expression, although the possibility that they are involved in the CSE induction by Cu10 cannot be excluded.

## 3. Discussions

**Figure 6:**
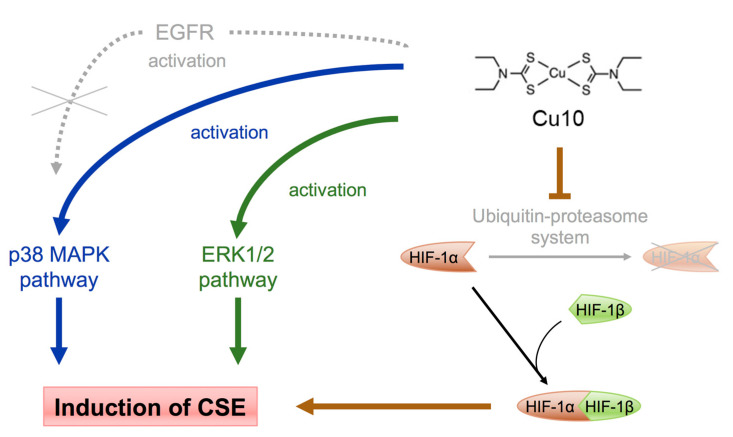
Intracellular signal pathways that mediate the transcriptional induction of CSE by Cu10 in vascular endothelial cells. Involvement of activation of the MAPK (ERK1/2 and p38 MAPK) and HIF-1α/HIF-1β pathways by Cu10 was shown in the present study, although the mechanisms underlying the activation remain to be determined.

Clarification of the intracellular signaling pathways that mediate expression of reactive sulfur species-producing enzymes in vascular endothelial cells provides important insights into cellular defense mechanisms against toxic heavy metals and oxidative stress. Vascular tissue is known to express CSE [41,42,43]. Although some reports have examined conditions that influence endothelial cell expression of CSE [29,30,31], the intracellular signaling pathways that mediate endothelial cell CSE expression have not been fully elucidated. In the present study, we have identified several pathways that mediate the induction of CSE transcription in cultured bovine aortic endothelial cells using Cu10 copper complex as a molecular probe. The following results were obtained: (1) Cu10 increased the transcription of CSE, but not that of other reactive sulfur species-producing enzymes, including CBS, 3-MST, and CARS2; (2) this induction required the complete Cu10 complex, i.e., the combination of diethyldithiocarbamate ligand and copper atom; and (3) transcriptional induction of CSE by Cu10 was mediated by the ERK1/2, p38 MAPK, and HIF-1α/HIF-1β pathways. The present study was undertaken, utilizing a bio-organometallics strategy, which revealed that three intracellular pathways—ERK1/2, p38 MAPK, and HIF-1α/HIF-1β—mediate the induction of CSE transcription in vascular endothelial cells (Figure 6). Additionally, although little is known about the transcription factors involved in the expression of reactive sulfur species-producing enzymes, the present study revealed that transcription factors p65, Sp1, and ATF4 are involved in the constitutive expression of CSE in endothelial cells, although the possibility that they are involved in the CSE induction by Cu10 cannot be excluded.

Several reports indicate that CSE expression is regulated via the ERK1/2 or p38 MAPK pathway in cells other than vascular endothelial cells. Specifically, CSE can be induced through the activation of the ERK1/2 pathway in human macrophages by lipopolysaccharide [44] by hydrogen peroxide in rat cardiomyocytes [45] and by glucose in mouse pancreatic islets [46]. CSE induction via p38 MAPK by lipopolysaccharide in rat macrophages [47] and by gliotoxin in rat glioma cells [48] has been shown. Partial involvement of the ERK1/2 and p38 MAPK pathways in induction of endothelial CSE has been demonstrated in the present study, suggesting that these two pathways are also involved in CSE induction in endothelial cells, as observed in other cell types described above. Cu10 activates Nrf2 [19] in vascular endothelial cells, suggesting that oxidative stress may increase in the cells. It is likely that the generated reactive oxygen is involved in the activation of intracellular signal pathways, such as the ERK1/2 and p38 MAPK pathways, although there could be alternative mechanisms. Additionally, the present study revealed the involvement of the HIF-1α/HIF-1β pathway in endothelial CSE induction. As we previously reported that the HIF-1α/HIF-1β pathway can mediate syndecan-4 induction in vascular endothelial cells [25], it is likely that the HIF-1α/HIF-1β pathway plays specific roles in regulating the expression of particular proteins in vascular endothelial cells; vascular endothelial cells directly contact the blood and may be sensitive to hypoxic conditions. Several other transcription factors—Nrf2 [49], NF-κB [44], Sp1 [46,50,51,52], and ATF4 [29,31]—have been identified as possible factors involved in inducing CSE in other cell types. The present study showed that these transcriptional factors are involved in constitutive CSE transcription but not in CSE induction by Cu10 in vascular endothelial cells. Although the PKA pathway may also mediate CSE expression [40], its involvement in CSE induction by Cu10 was not confirmed. Some intracellular signaling pathways that regulate the production of reactive sulfur species by CSE may be unique to vascular endothelial cells.

Cytoprotective roles have been reported for CSE-produced reactive sulfur species. For example, reactive sulfur species produced by CSE protect human fibroblasts from nickel-stimulation [53]. In vascular tissue, reactive sulfur species produced by CSE protect vascular endothelial cells [9] and myoblast cells [54] from cadmium cytotoxicity. Additionally, CSE overexpression protects against atherosclerosis progression [55], suggesting the importance of vascular CSE in preventing vascular lesions. These reports suggest that CSE expression is broadly responsible for defenses against toxic substances, especially heavy metals, and preventing vascular disorders. As both cadmium [6] and Cu10 [20] induce metallothionein, a cysteine-rich protein that protects against heavy metal toxicity, as do reactive sulfur species, in vascular endothelial cells, toxic heavy metal(s) and Cu10 may each have the ability to induce endothelial CSE to increase reactive sulfur species. The fact that cadmium [56], methylmercury [32], and Cu10 [26] activate p38 MAPK in vascular endothelial cells supports this hypothesis. Hyperhomocysteinemia, which is associated with complex multifactorial cardiovascular disorders, is related to altered endothelial cell redox balance [57] and the activation of intracellular signal pathways [58]. In addition, the transsulfuration pathway is closely linked with the production of intracellular anti-oxidant responses, including the mentioned metallothionein induction and the synthesis of a key antioxidant, glutathione [59], suggesting that Cu10, an inducer of endothelial metallothionein and CSE gene expression, may be a good tool for drug development. Studies using another molecular probe, which may be obtained from the other library of organic-inorganic hybrid molecules, should be performed to analyze mechanisms underlying the induction of endothelial CSE protein expression. Additionally, growth factors and cytokines that regulate vascular endothelial cell function via the activation of MAPK pathways may also be involved in the expression of reactive sulfur species-producing enzymes by the cells. In addition to bio-organometallics studies, further studies on endothelial cell CSE induction by toxic substances, such as heavy metals and regulatory factors, such as fibroblast growth factor-2 and transforming growth factor-β, should be performed to clarify cellular defense mechanisms against such substances.

## 4. Materials and Methods

### 4.1. Materials

Bovine aortic endothelial cells were purchased from Cell Applications (San Diego, CA, USA). Dulbecco’s modified Eagle’s medium (DMEM) and calcium- and magnesium-free phosphate buffered saline (CMF-PBS) were purchased from Nissui Pharmaceutical (Tokyo, Japan). Trypsin (1:250) Powder, Opti-Minimal essential medium (MEM)^®^ Reduced Serum Medium, Lipofectamine^®^ RNAiMAX Transfection Reagent, Lipofectamine^®^ LTX & Plus Reagent, the High-Capacity cDNA Reverse Transcription Kit, MagicMark™ XP Western Protein Standard, and NE-PER^®^ Nuclear and Cytoplasmic Extraction Reagents were purchased from Thermo Fisher Scientific (Waltham, MA, USA). Cell culture dishes were purchased from Corning (Corning, NY, USA). Fetal bovine serum (FBS), dimethyl sulfoxide, chloroform, acetic acid, hydrochloric acid, bromophenol blue, ammonium peroxodisulfate, copper sulfate, thapsigargin, Na01, mouse monoclonal anti β-actin antibody, and anti-glyceraldehyde-3-phosphate monoclonal antibody (peroxidase conjugated) were purchased from Wako Pure Chemical Industries (Osaka, Japan). Immobilon^®^-P Transfer Membranes were from Merck Millipore (Billerica, MA, USA). DynaMarker^®^ Protein MultiColor III protein standards were purchased from BioDynamics Laboratory (Tokyo, Japan). Absorbent paper was purchased from ATTO (Tokyo, Japan). QIAzol lysis reagent was purchased from QIAGEN (Venlo, Nederland). GeneAce SYBR^®^ qPCR Mix α was purchased from Nippon Gene (Tokyo, Japan). Anti-CSE rabbit polyclonal antibody was prepared as described previously [9], and anti-CBS mouse monoclonal antibody (M01) was obtained from Abnova Corporation (Taipei, Taiwan). Anti-MPST (3-MST) mouse monoclonal antibody (D-8), anti-CREB2 (ATF4) rabbit polyclonal antibody (C-20), and anti-lamin A/C mouse monoclonal antibody (636) were purchased from Santa Cruz Biotechnology (Santa Cruz, CA, USA). Anti-CARS2 rabbit polyclonal antibody (ARP68125P050_P050) was purchased from Aviva Systems Biology (San Diego, CA, USA). Anti-HIF-1α mouse monoclonal antibody (610958) was purchased from BD Biosciences (Franklin Lakes, NJ, USA). Anti-Nrf2 rabbit polyclonal antibody (NBP1-32822) was purchased from Novus Biologicals (Littleton, CO, USA). Phospho-p44/42 MAPK (Erk1/2)(Thr202/Tyr204) rabbit polyclonal antibody (#9101S), p44/42 MAPK (Erk1/2) rabbit polyclonal antibody (#9102S), phospho-p38 MAPK rabbit polyclonal antibody (#9211S), p38 MAPK rabbit polyclonal antibody (#9212S), phospho-SAPK/JNK mouse monoclonal antibody (#9255S), SAPK/JNK rabbit monoclonal antibody (#9252S), HRP-linked anti-rabbit IgG antibody (#7074S), and HRP-linked anti-mouse IgG antibody (#7076S) were purchased from Cell Signaling Technology (Danvers, MA, USA). The protein kinase A inhibitor H89, the epidermal growth factor receptor (EGFR) inhibitor PD153035, the ERK inhibitor PD98059, the p38 MAPK inhibitor SB203580, and the JNK inhibitor SP600125 were purchased from Cayman Chemical (Ann Arbor, MI, USA). The adenylyl cyclase inhibitor SQ22536 was purchased from R&D Systems (Minneapolis, MN, USA). Cu10, Fe05, Ni06, and Zn01 were from Tokyo Chemical Industry (Tokyo, Japan). Cu36, Cu38, Cu40, Cu42, Cu50, and Cu52 were synthesized employing previously described procedures [19,26,60]. Elemental ratios expected for Cu36 [C_10_H_16_CuN_2_S_4_]: C, 33.74; H, 4.53; Cu, 17.85; N, 7.87; S, 36.02, found: C, 33.53; H, 4.39; N, 7.87. Expected ratios for Cu38 [C_12_H_20_CuN_2_S_4_]: C, 37.53; H, 5.25; Cu, 16.54; N, 7.29; S, 33.39, found: C, 37.43; H, 5.12; N, 7.26. Expected ratios for Cu40 [C_10_H_16_CuN_2_O_2_S_4_]: C, 30.95; H, 4.16; Cu, 16.38; N, 7.22; O, 8.25; S, 33.05, found: C, 30.56; H, 3.93; N, 7.04. Expected ratios for Cu42 [C_12_H_22_CuN_4_S_4_]: C, 34.80; H, 5.35; Cu, 15.34; N, 13.53; S, 30.97, found: C, 34.50; H, 5.20; N, 13.39. Expected ratios for Cu50 [C_18_H_36_CuN_2_S_4_]: C, 45.78; H, 7.68; Cu, 13.45; N, 5.93; S, 27.15, found: C, 45.48; H, 7.74; N, 5.95. Expected ratios for Cu52 [C_14_H_28_CuN_2_S_4_]: C, 40.40; H, 6.78; Cu, 15.27; N, 6.73; S, 30.81, found: C, 38.05; H, 6.38; N, 6.45. The yields of Cu36, Cu38, Cu40, Cu42, Cu50, and Cu52 were 68.7%, 60.0%, 70.4%, 68.5%, 29.3%, and 58.3%, respectively. Other reagents were purchased from Nacalai Tesque (Kyoto, Japan).

### 4.2. Cell Culture and Treatments

Bovine aortic endothelial cells were cultured in a humidified atmosphere of 5% CO_2_ at 37 C in DMEM, supplemented with 10% FBS until confluency. Cells were transferred into 35-mm dishes and further cultured until confluent (dense culture) or transferred into 100-mm dishes at 1 × 10^4^ cells/cm^2^ and cultured for 24 h (sparse culture). The medium was then discarded, and the cells were used for the following experiments after washing twice with serum-free DMEM. Specifically, cells were exposed to Cu10 (5 or 10 µM) or other metal complexes (5 µM each) in serum-free DMEM for 4, 8, 12, or 24 h at 37 C. It was confirmed that there was no nonspecific cell damage by microscopic observation.

### 4.3. siRNA Transfection

Transfection of siRNAs was performed using Lipofectamine RNAiMAX, according to the manufacturer’s protocol. Briefly, annealed siRNA duplexes and Lipofectamine RNAiMAX were dissolved in Opti-MEM^®^ in separate tubes and incubated for 5 min at room temperature, followed by mixing and incubation for 20 min at room temperature. Bovine aortic endothelial cells were cultured in DMEM and supplemented with 10% FBS in 35-mm dishes until 70–80% confluency, followed by incubation at 37 C in fresh DMEM supplemented with 10% FBS in the presence of the siRNA/Lipofectamine RNAiMAX mixture. Final concentrations of siRNA and Lipofectamine RNAiMAX were 18 nM and 0.09%, respectively. After 4 h, the medium was changed to DMEM supplemented with 10% FBS, and the cells were incubated at 37 C for 20 h. The medium was then changed to DMEM and the cells were treated for 12 h with Cu10 (5 and 10 µM). The sequences of the sense and antisense strands of siRNAs are shown in Table 1.

### 4.4. Western Blot Analysis

Dense and sparse cultures of bovine aortic endothelial cells were lysed in sodium dodecyl sulfate sample buffer (50 mM Tris-HCl buffer, pH 6.8, containing 2% sodium dodecyl sulfate and 10% glycerol) and incubated at 95 C for 5 min. Separately, the nuclear fraction of bovine aortic endothelial cells was prepared using the NE-PER^®^ Nuclear and cytoplasmic extraction reagents, according to the manufacturer’s protocol. Protein concentrations were determined using a bicinchoninic acid protein assay reagent kit (Thermo Fisher Scientific). 2-Mercaptoethanol and bromophenol blue (1.67% each) were added to samples and incubated at 95 C for 3 min. Cellular proteins were separated by sodium dodecyl sulfate-polyacrylamide gel electrophoresis on 8%, 10%, or 12% polyacrylamide gels and electrotransferred onto polyvinylidene difluoride membranes (0.2 µm) at 2 mA/cm^2^ for 1 h. Membranes were blocked with 5% skim milk in 20 mM Tris-HCl buffer solution (pH 7.5), containing 15 mM NaCl and 0.1% Tween 20 (TTBS) or 2% serum albumin-TTBS solution, then incubated with a primary antibody at 4 C overnight. After washing with TTBS, membranes were incubated with horseradish peroxidase-conjugated secondary antibodies for 1 h at room temperature. Immunoreactive bands were visualized by enhanced chemiluminescence using Chemi-Lumi One L and scanned with a LAS 3000 Imager (Fujifilm, Tokyo, Japan).

### 4.5. Real-Time Reverse Transcription-Polymerase Chain Reaction (RT-PCR) Analysis

Dense and sparse cultures of bovine aortic endothelial cells were washed twice with CMF-PBS, then lysed with QIAzol lysis reagent. A quarter volume of chloroform was mixed with each lysate. Lysates were then centrifuged, and supernatants were harvested, followed by the addition of equal volumes of 70% ethanol. These suspensions were centrifuged at 15,000× *g*, and supernatants were discarded. Precipitates were resuspended in 70% ethanol and centrifuged at 15,000× *g*, followed by the collection and drying of the precipitates containing total RNA. Complementary DNA was synthesized from each mRNA sample using a high-capacity cDNA reverse transcription kit. Real-time PCR was performed using GeneAce SYBR qPCR mix α with 10 ng cDNA and primers (Table 2) in a StepOnePlus real-time PCR system. Levels of CSE, CBS, 3-MST, CARS2, HIF-1α, HIF-1β, p65, Sp1, ATF4, and β_2_-microgloblin (B2M) mRNAs were quantified using the relative standard curve method. The fold change in the intensity value of the target gene was normalized to that of B2M.

### 4.6. Statistical Analyses

Statistical analyses were performed with a student’s *t*-test or analysis of variance with a Dunnett’s or Tukey-Kramer’s test for the multiple comparison with Statcel3 (OMS, Tokyo, Japan). *P* values less than 0.05 was considered to indicate statistically significant differences.

## Figures and Tables

**Table 1:** Sequences of sense and antisense siRNA strands.

Gene	Sense (5′→3′)	Antisense (5′→3′)
HIF-1α	GGGAUUAACUCAGUUUGAACUdTdT	UUCAAACUGAGUUAAUCCCAUdTdT
HIF-1β	GAACUCUUAGGAAAGAAUAUUdTdT	UAUUCUUUCCUAAGAGUUCCUdTdT
Nrf2	CCAUUGAUCUCUCUGAUCUdTdT	AGAUCAGAGAGAUCAAUCGdTdT
p65	AUUGAAAGGGCUCUUUUUCAUdTdT	GAAAAAGAGCCCUUUCAAUGGdTdT
Sp1	GUUUAUAUAUACAUACAUAAU	UAUGUAUGUAUAUAUAAACUA
ATF4	AAUCAAACUCCUUCAAAUCdTdT	GAUUUGAAGGAGUUUGAUUdTdT
Negative control	UUCUCCGAACGUGUCACGUdTdT	ACGUGACACGUUCGGAGAAdTdT

**Table 2:** Bovine gene-specific primers for quantitative real-time PCR.

Gene	Sense (5′→3′)	Antisense (5′→3′)
CSE	TCTCTTGGAGCAGTTCCATCTCCTA	GCAGCCCAGGATAAATAACCTTTTC
CBS	GGACTCGGTGCGGAACTACA	GGCAACACGGTCAGCGG
3-MST	GCAGTGGGTGGCTGAGGC	CGATGTCAAAGAAGGCGGC
CARS2	GAGGCGACAGGTACGGCAAG	CAGACTGGCGATGGTGGAAC
HIF-1α	GCTTGCTCATCAGTTGCCAC	GCATCCAGAAGTTTCCTCACAC
HIF-1β	TAAGGAGCGGTTTGCCAGGTC	TTCTGTTATGTAGGCTGTCATCTTGTTC
p65	GATGGCTTCTATGAGGCTGAG	TTGTTGTTGGTCTGGATGC
Sp1	CTCTAAGCATCAGGAATCAGAAGTC	TCAGAAGCCCACACATCAAAG
ATF4	TGGTCTCAGACAACAGCAAG	AGCTCATCTGGCATGGTTTC
B2M	CCATCCAGCGTCCTCCAAAGA	TTCAATCTGGGGTGGATGGAA

## Supplementary Materials

Supplementary materials can be found at https://www.mdpi.com/1422-0067/21/17/6053/s1. Figure S1. The expression of CBS, 3-MST, and CARS2 in dense and sparse vascular endothelial cells after treatment with Cu10. Figure S2. Phosphorylation of ERK1/2, p38 MAPK, and JNK in vascular endothelial cells after treatment with Cu10. Figure S3. The expression of HIF1α protein in vascular endothelial cells treated with Cu10. Figure S4. The expression of Nrf2 protein in vascular endothelial cells treated with Cu10. Figure S5. The expression of ATF4 protein in the nuclear fraction of vascular endothelial cells treated with Cu10.

## Abbreviations

ATF4	Activating transcription factor 4
B2M	β_2_-microgloblin
CARS2	Cysteinyl-tRNA synthetase 2
CBS	Cystathionine beta-synthase
CSE	Cystathionine gamma-lyase
Cu10	Copper (II) bis(diethyldithiocarbamate)
Cu36	Bis(1-pyrrolidinecarbodithioato)copper (II)
Cu38	Bis(1-piperidinecarbodithioato)copper (II)
Cu40	Bis(4-morpholinecarbodithioato)copper (II)
Cu42	Bis(4-methylpiperazine-1-carbodithioato)copper (II)
Cu50	Bis(diisobutyldithiocarbamato)copper (II)
Cu52	Bis(butylethyldithiocarbamato)copper (II)
EGFR	Epidermal growth factor receptor
ERK	Extracellular signal-regulated kinase
Fe05	Iron(III) tris(diethyldithiocarbamate)
GAPDH	Glyceraldehyde-3-phosphate dehydrogenase
HIF-1	Hypoxia-inducible factor-1
JNK	c-Jun N-terminal kinase
MAPK	Mitrogen-activated phosphate kinase
3-MST	3-mercaptopyruvate sulfertransferase
Na01	Sodium diethyldithiocarbamate trihydrate
Ni06	Nickel(II) bis(diethyldithiocarbamate)
PKA	Protein kinase A
NF-κB	Nuclear factor-kappa B
Nrf2	NF-E2-related factor 2
RSS	Reactive sulfur species
Sp1	Specificity protein-1
TTBS	Tween tris buffered saline
Zn01	Zinc(II) bis(diethyldithiocarbamate)
