# Supplementary material for: Transcriptional Induction of Cystathionine γ-Lyase, a Reactive Sulfur-Producing Enzyme, by Copper Diethyldithiocarbamate in Cultured Vascular Endothelial Cells
Source: Int J Mol Sci. 2020 Aug 22;21(17):6053. doi: 10.3390/ijms21176053 (PMC7503448; doi:10.3390/ijms21176053)
Supplement: Supplementary file 1 [file ijms-21-06053-s001.pdf]

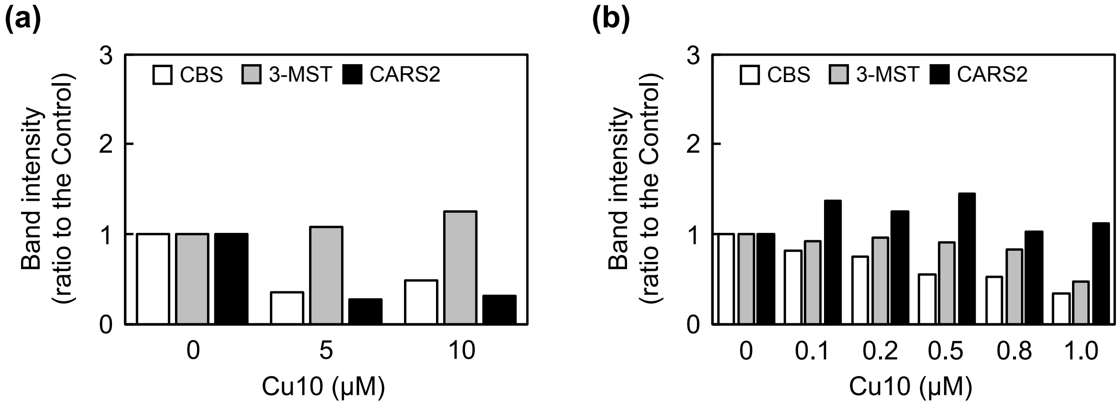

Figure S1. The expression of CBS, 3-MST, and CARS2 in **(a)** dense and **(b)** sparse vascular endothelial cells after treatment with Cu10. The ratio of the intensity of CBS, 3-MST, and CARS2 in Figure 1D to those of GAPDH; values are means of two replicates from two independent experiments.

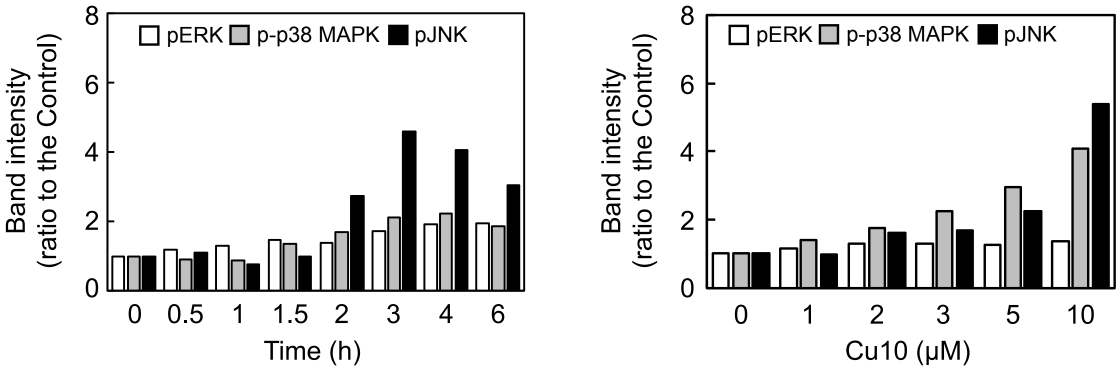

Figure S2. Phosphorylation of ERK1/2, p38 MAPK, and JNK in vascular endothelial cells after treatment with Cu10. The ratio of the intensity of p-ERK1/2, p-p38 MAPK, and p-JNK in Figure 3A to those of ERK1/2, p38 MAPK, and JNK, respectively; values are means of two replicates from two independent experiments.

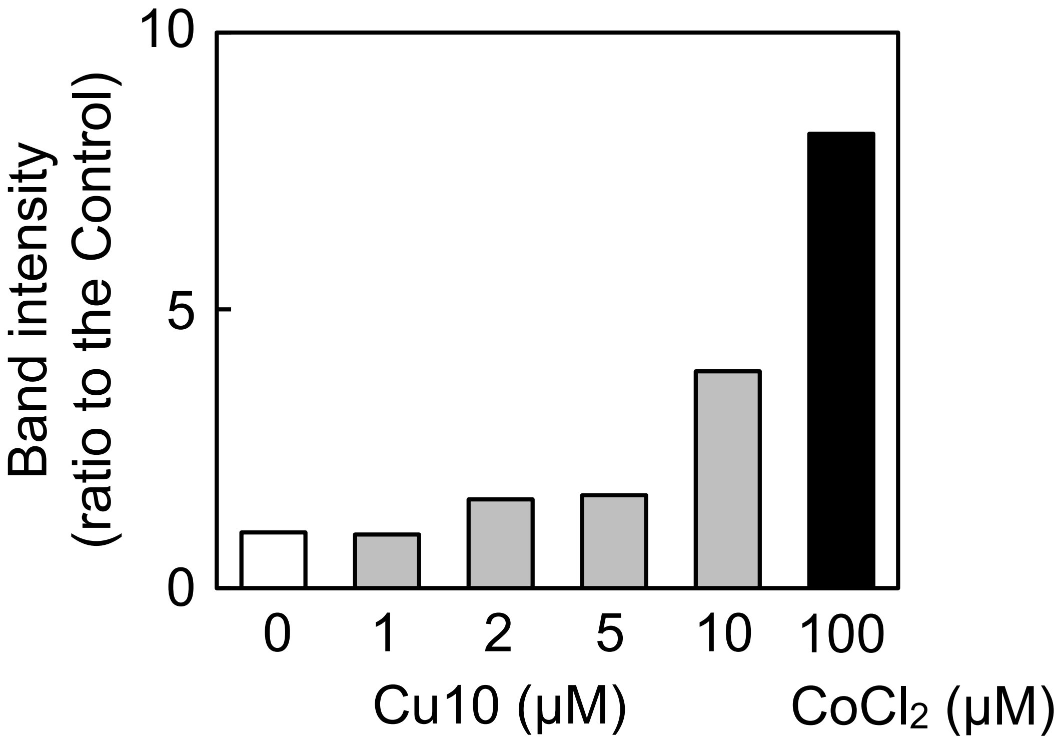

Figure S3. The expression of HIF1α protein in vascular endothelial cells treated with Cu10. The ratio of the intensity of HIF-1α in Figure 4A to that of GAPDH; values are means of two replicates from two independent experiments.

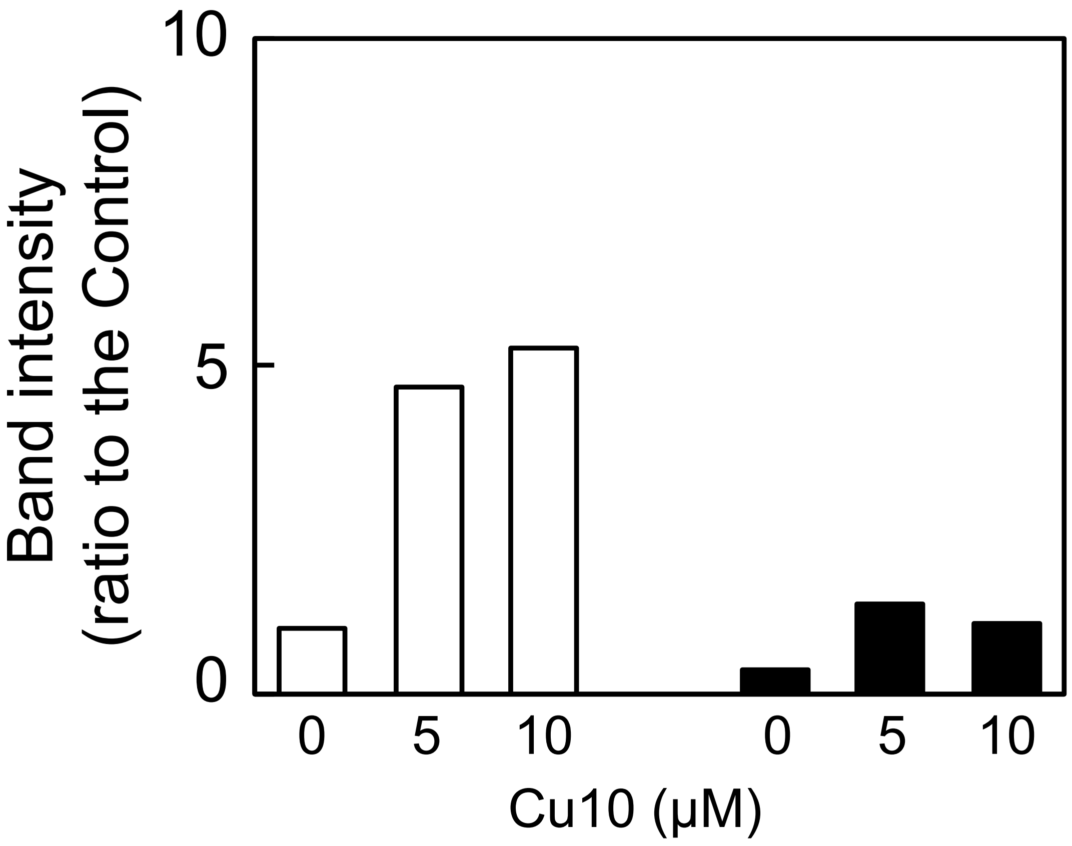

Figure S4. The expression of Nrf2 protein in vascular endothelial cells treated with Cu10. The ratio of the intensity of Nrf2 in Figure 5B to that of β-actin; values are means of two replicates from two independent experiments.

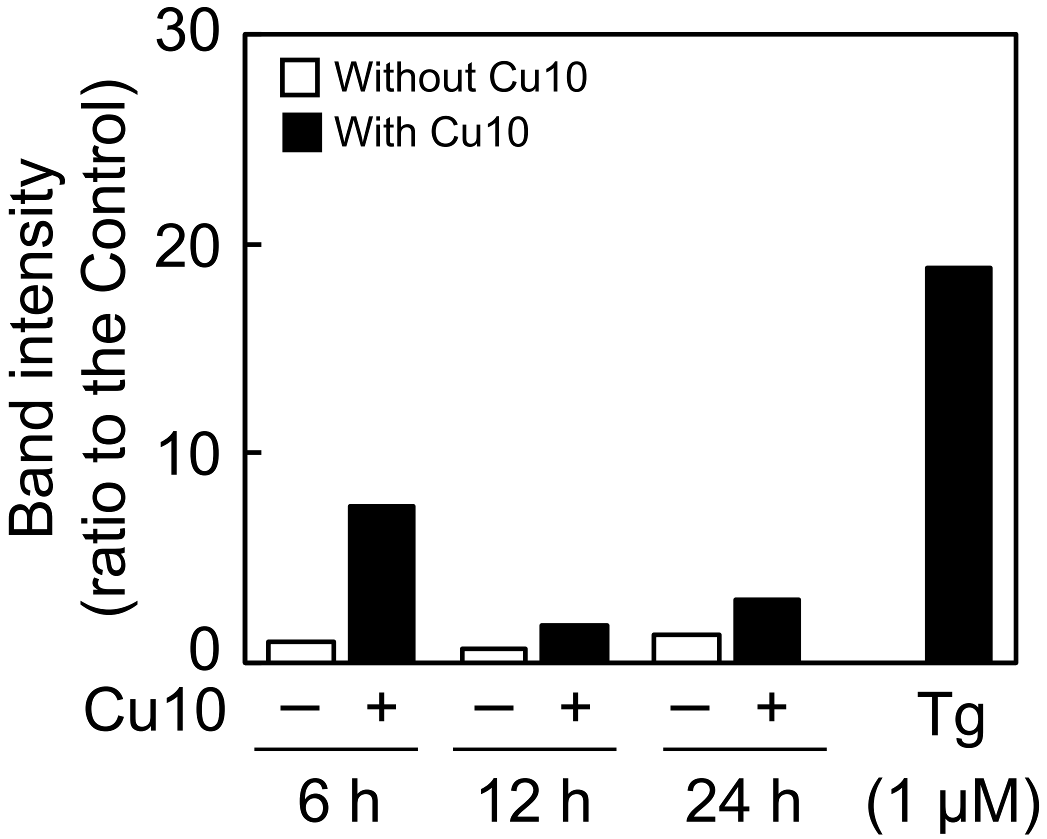

Figure S5. The expression of ATF4 protein in the nuclear fraction of vascular endothelial cells treated with Cu10. The ratio of the intensity of ATF4 in Figure 5E to that of Lamin A/C; values are means of two replicates from two independent experiments.
